# Comprehensive analysis of *ID* genes reveals the clinical and prognostic value of *ID3* expression in acute myeloid leukemia using bioinformatics identification and experimental validation

**DOI:** 10.1186/s12885-022-10352-6

**Published:** 2022-11-29

**Authors:** Qi Zhao, Yun Wang, Di Yu, Jia-Yan Leng, Yangjing Zhao, Mingqiang Chu, Zijun Xu, Hao Ding, Jingdong Zhou, Tingjuan Zhang

**Affiliations:** 1 Department of Hematology, Affiliated People’s Hospital of Jiangsu University, 8 Dianli Rd, 212002 Zhenjiang, Jiangsu P. R. China; 2 Zhenjiang Clinical Research Center of Hematology, 212002 Zhenjiang, Jiangsu P. R. China; 3 The Key Lab of Precision Diagnosis and Treatment of Zhenjiang City, Jiangsu 212002 Zhenjiang, P. R. China; 4 Laboratory Center, Affiliated People’s Hospital of Jiangsu University, 212002 Zhenjiang, Jiangsu P. R. China; 5 Department of Respiratory Disease, Affiliated People’s Hospital of Jiangsu University, 8 Dianli Rd, 212002 Zhenjiang, Jiangsu P. R. China; 6 Department of Laboratory Medicine, School of Medicine, Jiangsu University, 212013 Zhenjiang, Jiangsu P. R. China; 7 Department of Oncology, Affiliated People’s Hospital of Jiangsu University, 8 Dianli Rd, 212002 Zhenjiang, Jiangsu P. R. China

**Keywords:** AML, Expression, *ID*, *ID3*, Prognosis

## Abstract

**Background:**

Dysregulation of inhibitor of differentiation/DNA binding (*ID*) genes is linked to cancer growth, angiogenesis, invasiveness, metastasis and patient survival. Nevertheless, few investigations have systematically determined the expression and prognostic value of *ID* genes in acute myeloid leukemia (AML).

**Methods:**

The expression and clinical prognostic value of *ID* genes in AML were first identified by public databases and further validated by our research cohort.

**Results:**

Using public data, the expression of *ID1*/*ID3* was markedly downregulated in AML, and the expression of *ID2* was greatly upregulated in AML, whereas *ID4* showed no significant difference. Among the *ID* genes, only *ID3* expression may be the most valuable prognostic biomarker in both total AML and cytogenetically normal AML (CN-AML) and especially in CN-AML. Clinically, reduced *ID3* expression was greatly associated with higher white blood cell counts, peripheral blood/bone marrow blasts, normal karyotypes and intermediate cytogenetic risk. In addition, low *ID3* expression was markedly related to *FLT3* and *NPM1* mutations as well as wild-type *TP53*. Despite these associations, multivariate Cox regression analysis revealed that *ID3* expression was an independent risk factor affecting overall survival (OS) and disease free survival (DFS) in CN-AML patients. Biologically, a total of 839 mRNAs/lncRNAs and 72 microRNAs were found to be associated with *ID3* expression in AML. Importantly, the expression of *ID3* with discriminative value in AML was further confirmed in our research cohort.

**Conclusion:**

The bioinformatics analysis and experimental verification demonstrate that low *ID3* expression independently affects OS and DFS in patients with CN-AML, which might be seen as a potential prognostic indicator in CN-AML.

**Supplementary Information:**

The online version contains supplementary material available at 10.1186/s12885-022-10352-6.

## Background

Acute myeloid leukemia (AML) is a clonal disease characterized by amplification of immature myeloid progenitors with differentiation arrest in the bone marrow (BM), finally resulting in hematopoietic failure [1]. AML is a clinically, cytogenetically and molecularly heterogeneous disease with variable clinical outcomes [1]. The features of morphology, immunology, cytogenetics and molecular biology (MICM) are the basis for AML diagnosis [1]. Cytogenetic abnormalities also provide the most important prognostic information of AML [2]. Molecular biological alterations, such as gene mutations and aberrant gene expression, also play important roles in leukemogenesis and predict treatment response and patient survival [2]. Therefore, the identification of biological markers to develop a better prognostic, diagnostic and therapeutic risk stratification for AML is of great importance.

The inhibitor of differentiation/DNA binding (*ID*) genes (*ID1*/*ID2*/*ID3*/*ID4*) encode ID proteins that are transcriptional regulators controlling the timing of cell fate determination and differentiation in stem and progenitor cells during physiological development [3, 4]. It was suggested that ID proteins could have key roles in cancer development [3, 4]. At the same time, dysregulated *ID* gene expression was linked to tumor growth, invasiveness, metastasis, angiogenesis and patient survival [3, 4]. *ID1* and *ID2* overexpression has been shown to correlate with enhanced malignant potential in various types of cancers including AML [3, 4]. Although increased *ID1* expression was observed in AML patients, the prognostic value of *ID1* overexpression remains controversial [5–7]. In addition, the prognostic effect of *ID2* overexpression in AML was reported in our previous study [8]. In contrast, *ID4* functioned as a tumor suppressor presenting a paradigm shift in the context of *ID1* and *ID2* during the process of tumorigenesis and leukemogenesis [3]. *ID4* hypermethylation was an independent factor that affected clinical outcome and predicted leukemic transformation in patients with myelodysplastic syndrome (MDS) [9]. However, the function of *ID3* and its expression pattern in AML are not completely understood. Herein, we systematically explored the expression and clinical implications of *ID* genes expression in AML.

## Materials and methods

### Patients from public datasets and our hospital

The identification cohort included 173 AML patients with *ID* gene (*ID1*/*ID2*/*ID3*/*ID4*) expression data (RNA-Seq V2 data) from public The Cancer Genome Atlas (TCGA) datasets [10]. All AML patients received standard chemotherapy as induction therapy. Following induction therapy, 100 patients underwent chemotherapy only, whereas the remaining 73 patients underwent auto/allo-hematopoietic stem cell transplantation as consolidation treatment. The *ID* gene expression in AML compared with controls was analyzed by GEPIA [11].

The validation cohort contained 107 AML patients treated at the Affiliated People’s Hospital of Jiangsu University. Patients with antecedent hematological diseases or therapy-related AML were excluded. The clinical characteristics of the AML patients are shown in Supplementary Table S1. BM samples were collected from AML patients once they were diagnosed. A total of 32 healthy donors served as normal controls. The age of the AML patients (median 57, range 18–87) showed no significant differences from that of the controls (median 52, range 20–66) (*P* > 0.05). This current study was approved by the Ethics Committee of the Affiliated People’s Hospital of Jiangsu University, and all the individuals provided written informed consent.

### RNA isolation and reverse transcription

BM mononuclear cells (BMMNCs) were separated through gradient centrifugation using Lymphocyte Separation Medium (Solarbio, Beijing, China), and then used for total RNA extraction by TRIzol reagent (Invitrogen, Carlsbad, CA). Reverse transcription was performed to synthesize cDNA as reported [12–14].

### RT–qPCR

The detection of *ID3* and *ABL1* (housekeeping gene) mRNA was determined by real-time quantitative PCR (RT–qPCR) using AceQ qPCR SYBR Green Master Mix (Vazyme, Piscataway, NJ). The primers applied for *ID3* expression detection were 5’-ACTCAGCTTAGCCAGGTGGA-3’ (forward) and 5’-AAGCTCCTTTTGTCGTTGGA-3’ (reverse), whereas those for *ABL1* expression detection were 5’-TCCTCCAGCTGTTATCTGGAAGA-3’ (forward) and 5’-TCCAACGAGCGGCTTCAC-3’ (reverse). The relative *ID3* mRNA level was measured according to the 2^−∆∆Ct^ method [12–14].

### Bioinformatics analysis

All procedures regarding the bioinformatics analysis were carried out as described in our previous studies [15, 16]. To obtain the differentially expressed genes/miRNAs (DEGs/DEmiRs), analysis of the RNA sequencing data was conducted based on the raw read counts with the R/Bioconductor package “edgeR”. All statistical analyses were controlled for the false discovery rate (FDR) by the Benjamini–Hochberg procedure.

### Statistical analysis

Statistical analysis was carried out based on the SPSS 20.0 and GraphPad 5.0 software. Comparisons of continuous and categorical variables were conducted using the Mann–Whitney U test/Kruskal–Wallis test and Pearson’s χ2 test/Fisher’s exact test, respectively. Kaplan–Meier analysis (log-rank test) and Cox regression (proportional hazards model, backward method) were used to analyze the effect of *ID1*/*ID2*/*ID3*/*ID4* expression on survival including disease-free survival (DFS) and overall survival (OS). The ability of *ID3* expression to discriminate in AML patients from controls was evaluated by the receiver operating characteristic (ROC) curve and area under the ROC curve (AUC). A two-sided *P* value less than 0.05 was considered statistically significant in all analyses.

## Results

### Identification of reduced ID3 expression among ID genes correlated with prognosis in AML from public TCGA datasets

**Fig. 1:**
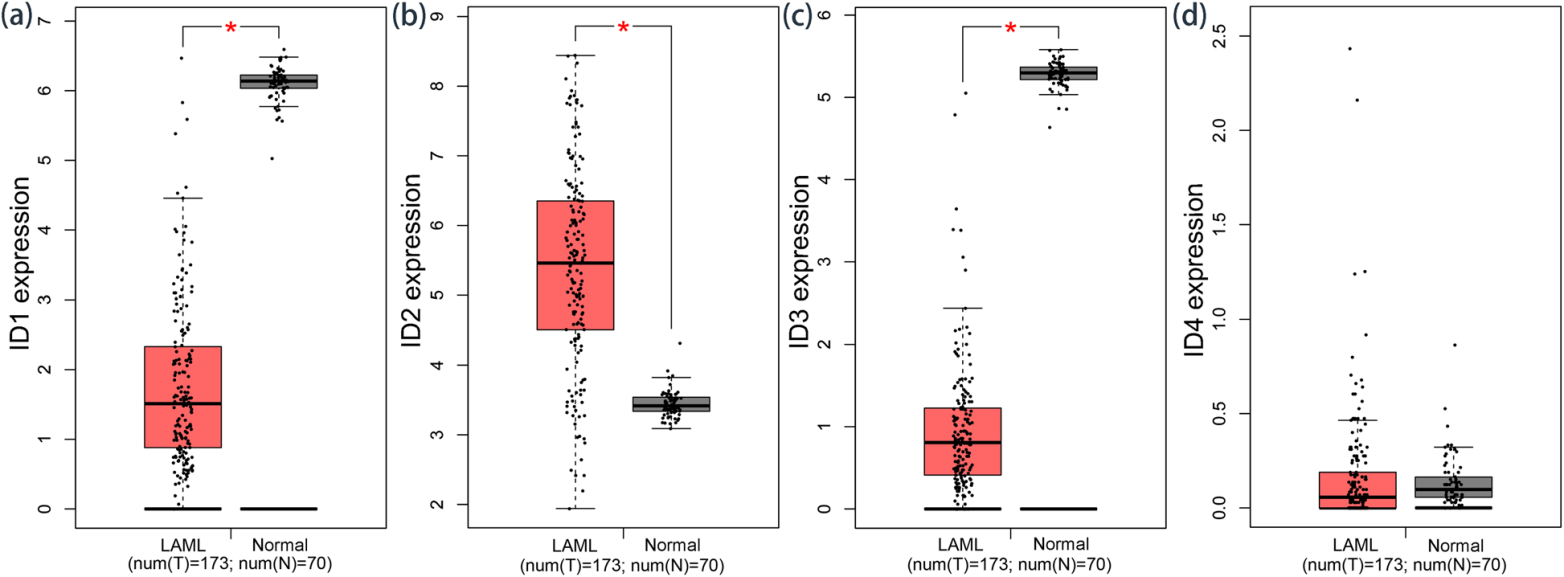
Expression of *ID* genes in AML. **a** *ID1* expression in AML; **b** *ID2* expression in AML; **c** *ID3* expression in AML; **d** *ID4* expression in AML. ^*^: *P* < 0.001. The expression of *ID* genes expression in AML compared with controls is analyzed in GEPIA (http://gepia.cancer-pku.cn/)

We first searched GEPIA to determine the expression of *ID* genes (*ID1*/*ID2*/*ID3*/*ID4*) in AML. As presented in Fig. 1, the expression of *ID1* and *ID3* was markedly downregulated (both *P* < 0.001), and the expression of *ID2* was greatly upregulated in AML (*P* < 0.001), whereas *ID4* showed no dramatic difference in expression (*P* > 0.05).

**Fig. 2:**
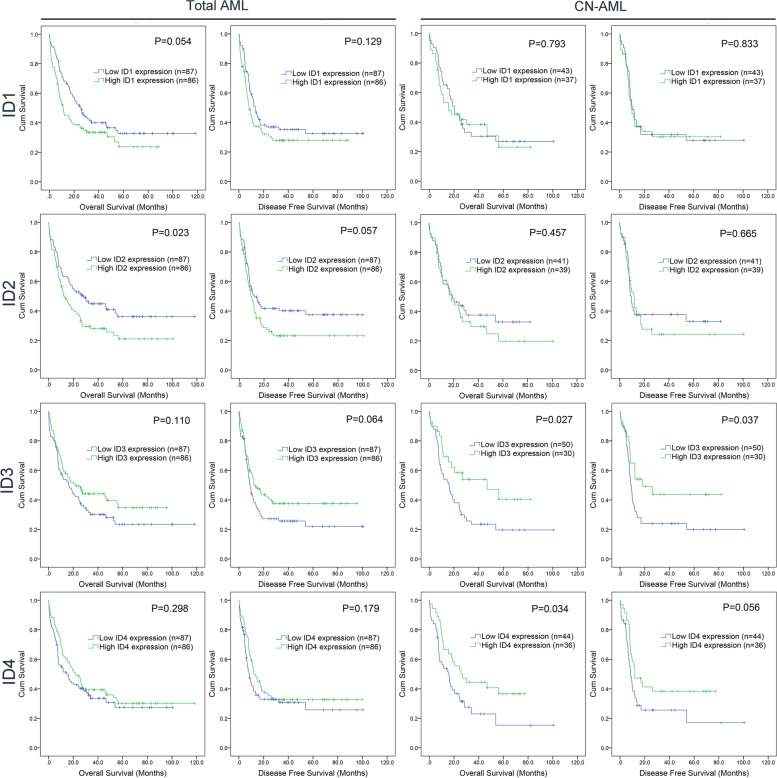
Prognostic value of *ID* genes in AML. The prognostic effect of *ID* genes (*ID1*/*ID2*/*ID3*/*ID4*) on overall survival and disease free survival were analyzed by Kaplan–Meier method

Next, to investigate the prognostic significance of the *ID* genes in AML, we evaluated the impact of *ID* gene expression on OS and DFS times by Kaplan–Meier analysis. When analyzing the prognostic value, the AML patients were divided into two groups by the median level of *ID* gene expression. In all the AML patients, only high *ID2* expression was markedly associated with a shorter OS time (*P* = 0.023), whereas the other *ID* members did not affect either OS or DFS time (*P* > 0.05) (Fig. 2). In cytogenetically normal AML (CN-AML) patients, lower *ID3* and *ID4* expression was nearly or markedly correlated with shorter OS (*P* = 0.027 and 0.034, respectively) and DFS (*P* = 0.037 and 0.056, respectively), whereas the other *ID* members did not affect either OS or DFS times (*P* > 0.05) (Fig. 2).

Finally, we further investigated the impact of *ID* gene expression on OS and DFS times in AML by Cox regression analysis. In all the AML patients, the expression of *ID1*, *ID2* and *ID3* independently affected the OS time (*P* = 0.016, 0.039 and 0.028, respectively) (Table 1), whereas the expression of *ID1* and *ID3* independently affected the DFS time (*P* = 0.043 and 0.022, respectively) (Supplementary Table S2). Among CN-AML patients, only *ID3* expression independently affected both the OS and DFS times (*P* = 0.030 and 0.041, respectively) (Table 1 and Supplementary Table S2).

**Table 1:** Cox regression univariate and multivariate analysis of variables for overall survival in AML patients

Variables	Univariate analysis		Multivariate analysis	
	Hazard ratio (95% CI)	*P* value	Hazard ratio (95% CI)	*P* value
Total AML				
* ID1* expression	1.433 (0.991–2.073)	0.056	1.616 (1.094–2.387)	0.016
* ID2* expression	1.530 (1.056–2.216)	0.025	1.477 (1.020–2.141)	0.039
* ID3* expression	0.741 (0.512–1.072)	0.112	0.646 (0.437–0.953)	0.028
* ID4* expression	0.823 (0.569–1.189)	0.300	-	-
CN-AML				
* ID1* expression	1.074 (0.627–1.840)	0.794	-	-
* ID2* expression	1.224 (0.717–2.088)	0.459	-	-
* ID3* expression	0.516 (0.284–0.939)	0.030	0.516 (0.284–0.939)	0.030
* ID4* expression	0.555 (0.319–0.964)	0.037	0.629 (0.356–1.109)	0.109

Multivariate analysis includes variables with *P* < 0.200 in univariate analysis

*AML* Acute myeloid leukemia, *CN-AML* Cytogenetically normal AML, *CI* Confidence interval

Taken together, these results suggest that *ID3* expression may be most valuable prognostic biomarker among the *ID* genes in AML, especially CN-AML, and it was selected for further analysis.

### Clinical significance of ID3 expression and its correlation with gene mutations in AML

**Fig. 3:**
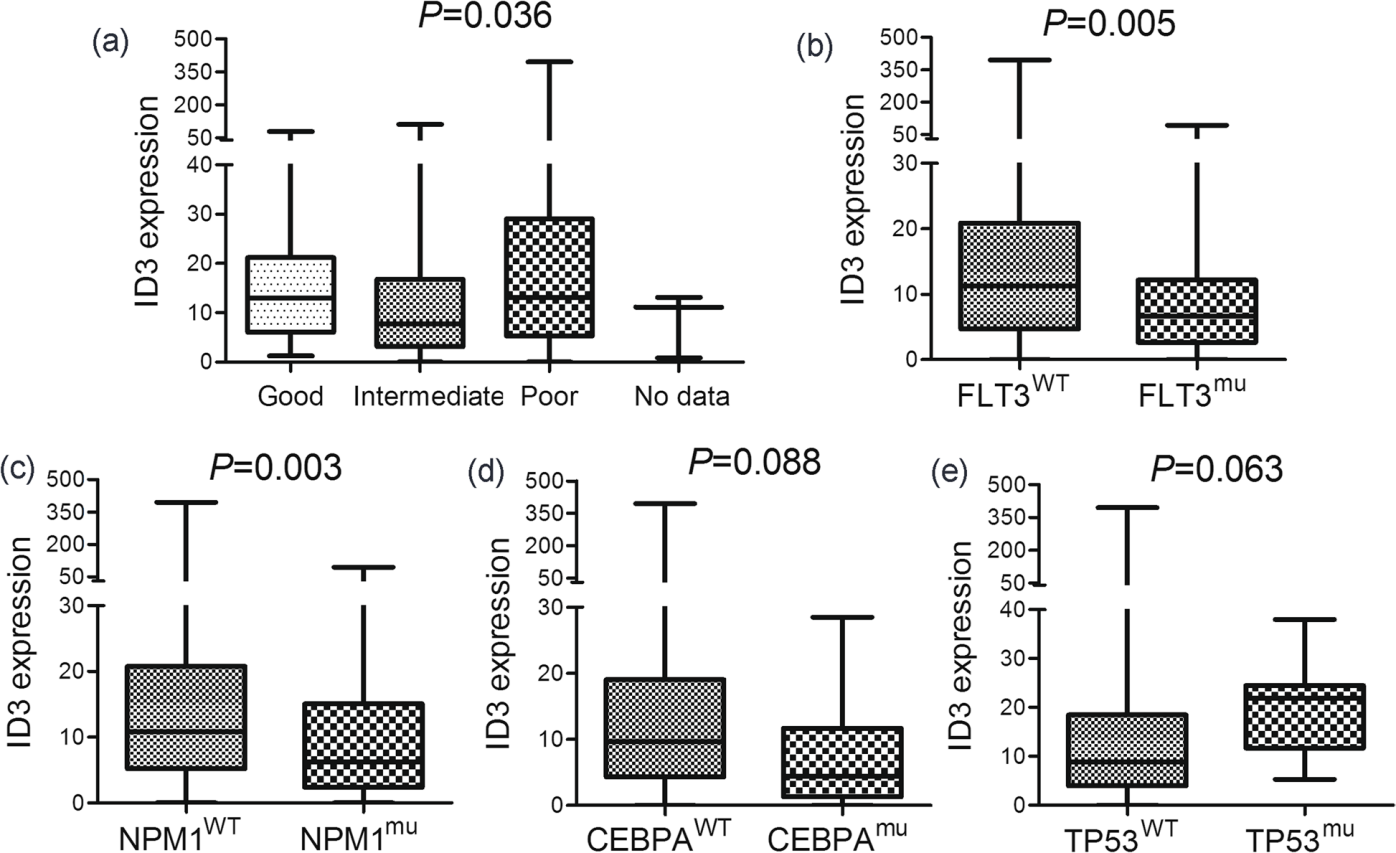
The associations of *ID3* expression with cytogenetic risks/genetic abnormalities in AML. **a** *ID3* expression among different cytogenetic risks of AML. **b** *ID3* expression in AML patients with and without *FLT3* mutations. **c** *ID3* expression in AML patients with and without *NPM1* mutations. **d** *ID3* expression in AML patients with and without *CEBPA* mutations. **e** *ID3* expression in AML patients with and without *TP53* mutations

To further analyze the clinical relevance of *ID3* expression in AML, the AML patients from TCGA dataset were divided into two groups by the median *ID3* expression level. Comparisons of clinicopathological features, including age, sex, white blood cell (WBC) count, peripheral blood (PB)/BM blasts, French–American–British (FAB) classifications, cytogenetics and gene mutations, between the two groups (low and high *ID3* expression) in both the total AML and the CN-AML cohort are shown in Table 2. In all AML patients, low *ID3* expression was greatly correlated with higher WBC counts and PB/BM blasts (*P* < 0.001, = 0.001 and = 0.002, respectively). Moreover, low *ID3* expression was markedly correlated with normal karyotype and intermediate cytogenetic risk (*P* = 0.004 and 0.014, respectively). Based on the results, we further compared *ID3* expression between AML patients with different cytogenetic risks, and confirmed the significant differences (*P* = 0.036, Fig. 3a). In addition, low *ID3* expression was markedly associated with *FLT3* and *NPM1* mutations as well as wild-type *TP53* (*P* = 0.018, 0.011 and 0.028, respectively). Similarly, we further determined *ID3* expression between AML patients with and without these gene mutations. Expectedly, marked differences were observed in subgroups divided by *FLT3* and *NPM1* status (*P* = 0.005 and 0.003, respectively, Fig. 3b-c), whereas a trend was observed in subgroups divided by *TP53* and *CEBPA* status (*P* = 0.063 and 0.088, respectively, Fig. 3d-e). In CN-AML, the above significant differences were not observed (Table 2).

**Table 2:** Correlation of *ID3* expression with clinic-pathologic characteristics in AML patients

Patient’s parameters	Total AML			CN-AML		
	Low (*n*=87)	High (*n*=86)	*P*	Low (*n*=50)	High (*n*=30)	*P*
Sex, male/female	44/43	49/37	.447	23/27	14/16	1.000
Median age, years (range)	57(18-82)	59 (21-88)	.727	57 (21-82)	59 (21-88)	.970
Median WBC, ×10^9^/L (range)	37 (1.4-297.4)	5.5 (0.4-171.9)	.000	50.5 (1.4-297.4)	8.6 (0.6-115.4)	.000
Median PB blasts, % (range)	49 (0-98)	17 (0-97)	.001	49 (0-98)	18.5 (0-97)	.137
Median BM blasts, % (range)	77 (32-100)	65.5 (30-100)	.002	76.5 (32-100)	71 (30-100)	.161
FAB classifications			.162			.370
M0	6	10	.307	1	2	.553
M1	24	20	.601	14	10	.624
M2	19	19	1.000	14	6	.595
M3	5	11	.124	0	1	.375
M4	20	14	.339	11	5	.774
M5	12	6	.212	10	4	.552
M6	0	2	.246	0	0	-
M7	0	3	.121	0	1	.375
No data	1	1	1.000	0	1	.375
Karyotypes			.342	-	-	-
normal	50	30	.004	-	-	-
t(15;17)	5	10	.188	-	-	-
t(8;21)	3	4	.720	-	-	-
inv(16)	4	6	.535	-	-	-
+8	4	4	1.000	-	-	-
del(5)	0	1	.497	-	-	-
-7/del(7)	2	5	.278	-	-	-
11q23	1	2	.621	-	-	-
others	6	8	.590	-	-	-
complex	11	14	.524	-	-	-
No data	1	2	.621	-	-	-
Risks (cytogenetic)			.069			
Good	12	20	.121	-	-	-
Intermediate	59	42	.014	-	-	-
Poor	15	22	.199	-	-	-
No data	1	2	.621	-	-	-
Risks (molecular)			.265			.061
Good	12	21	.084	0	1	.497
Intermediate	51	41	.172	45	29	.402
Poor	23	22	1.000	5	0	.151
No data	1	2	.621	0	0	-
Gene mutations						
*FLT3*^a^ (+/-)	32/55	17/69	.018	22/28	9/21	.244
*NPM1* (+/-)	32/55	16/70	.011	29/21	14/16	.361
*DNMT3A* (+/-)	24/63	18/68	.376	18/32	11/19	1.000
*IDH2* (+/-)	7/80	10/76	.456	5/45	5/25	.489
*IDH1* (+/-)	6/81	10/76	.307	3/47	6/24	.073
*TET2* (+/-)	8/79	7/79	1.000	5/45	4/26	.722
*RUNX1* (+/-)	4/83	5/81	.747	2/48	5/25	.096
*TP53* (+/-)	3/84	11/75	.028	0/50	1/29	.375
*NRAS* (+/-)	5/82	7/79	.566	4/46	3/27	1.000
*CEBPA*^b^ (+/-)	10/77	3/83	.080	7/43	1/29	.247
*WT1* (+/-)	7/80	3/83	.329	4/46	2/28	1.000
*PTPN11* (+/-)	5/82	3/83	.720	4/46	1/29	.645
*KIT* (+/-)	2/85	5/81	.278	0/50	0/30	-
*U2AF1* (+/-)	2/85	5/81	.278	1/49	0/30	1.000
*KRAS* (+/-)	3/84	4/82	.720	2/48	1/29	1.000
*ASXL1* (+/-)	0/87	3/83	.121	0/50	1/29	.375

Cytogenetic and molecular risk classifications are based on the 2017 European LeukemiaNet (ELN) classification. Patients without required information for FAB subtypes, karyotypes and molecular/cytogenetic risks are considered as no data

*AML* Acute myeloid leukemia, *WBC* White blood cells, *PB* Peripheral blood, *BM* Bone marrow, *FAB* French-American-British classification

^a ^*FLT3* mutation indicates both *FLT3-ITD* (high and low ratios) and *FLT3-TKD* mutations

^b ^*CEBPA* mutation indicates both mono- and bi-allelic *CEBPA* mutation

### The independent prognostic value of ID3 expression in AML

Because a marked correlation was found between *ID3* expression and common prognostic factors such as WBC, cytogenetics and gene mutations, we performed multivariate analysis by Cox regression to confirm the independent prognostic impact of *ID3* expression in AML after adjusting for the prognosis-related factors. Multivariate Cox regression analysis indicated that *ID3* expression was an independent risk factor affecting OS (*P* = 0.022, Table 3) and DFS (*P* = 0.043 and Supplementary Table S3) in CN-AML patients.

**Table 3:** Cox regression multivariate analysis of variables for overall survival in AML patients

Variables	Total AML				CN-AML			
	Univariate analysis		Multivariate analysis		Univariate analysis		Multivariate analysis	
	HR (95% CI)	*P*	HR (95% CI)	*P*	HR (95% CI)	*P*	HR (95% CI)	*P*
Age	1.040 (1.025–1.055)	< 0.001	1.028 (1.012–1.044)	< 0.001	1.023 (1.005–1.042)	0.014	1.012 (0.991–1.034)	0.272
WBC	1.005 (1.001–1.008)	0.018	1.007 (1.003–1.011)	0.002	1.005 (1.000-1.010)	0.032	1.003 (0.998–1.009)	0.223
Molecular risks	1.921 (1.487–2.481)	< 0.001	2.184 (1.609–2.963)	< 0.001	1.817 (0.760–4.344)	0.179	1.552 (0.573–4.201)	0.387
Treatment regimen	0.519 (0.355–0.761)	0.001	0.426 (0.275–0.661)	< 0.001	0.525 (0.306–0.902)	0.020	0.424 (0.240–0.746)	0.003
*FLT3* mutation^a^	1.325 (0.885–1.984)	0.171	1.642 (1.059–2.546)	0.027	1.418 (0.818–2.458)	0.214	-	-
*NPM1* mutation	1.155 (0.770–1.732)	0.486	-	-	1.059 (0.620–1.809)	0.833	-	-
*TP53* mutation	4.100 (2.291–7.339)	< 0.001	2.513 (1.288–4.903)	0.007	2.614 (0.354–19.301)	0.346	-	-
*CEBPA* mutation^b^	0.928 (0.470–1.834)	0.831	-	-	1.011 (0.432–2.365)	0.980	-	-
*ASXL1* mutation	2.365 (0.749–7.473)	0.142	3.634 (1.091–12.102)	0.036	10.795 (1.328–87.751)	0.026	19.441 (2.200-171.761)	0.008
*RUNX1* mutation	1.069 (0.761–1.051)	0.702	-	-	1.271 (0.502–3.214)	0.613	-	-
*ID1* expression	1.433 (0.991–2.073)	0.056	1.301 (0.853–1.985)	0.222	1.074 (0.627–1.840)	0.794	-	-
*ID2* expression	1.530 (1.056–2.216)	0.025	1.050 (0.690–1.598)	0.819	1.224 (0.717–2.088)	0.459	-	-
*ID3* expression	0.741 (0.512–1.072)	0.112	0.712 (0.465–1.090)	0.118	0.516 (0.284–0.939)	0.030	0.502 (0.278–0.906)	0.022
*ID4* expression	0.823 (0.569–1.189)	0.300	-	-	0.555 (0.319–0.964)	0.037	0.492 (0.263–0.922)	0.027

Variables including age (continuous variables), WBC (continuous variables), treatment regimen (with transplantation vs. without transplantation), molecular risks (good, intermediate, poor and unknown; classified by the 2017 European LeukemiaNet classification), FLT3/NPM1/TP53 mutation (wild type vs. mutant) and ID1/2/3/4 expression (low vs. high). Multivariate analysis includes variables with *P* < 0.200 in univariate analysis

*AML* Acute myeloid leukemia, *CN-AML* Cytogenetically normal AML, *HR* Hazard ratio, *CI* Confidence interval, *WB*C White blood cells

^a ^*FLT3* mutation indicates both *FLT3-ITD* (high and low ratios) and *FLT3-TKD* mutations

^b ^*CEBPA* mutation indicates both mono- and bi-allelic *CEBPA* mutation

### Molecular signatures correlated with ID3 expression in AML

**Fig. 4:**
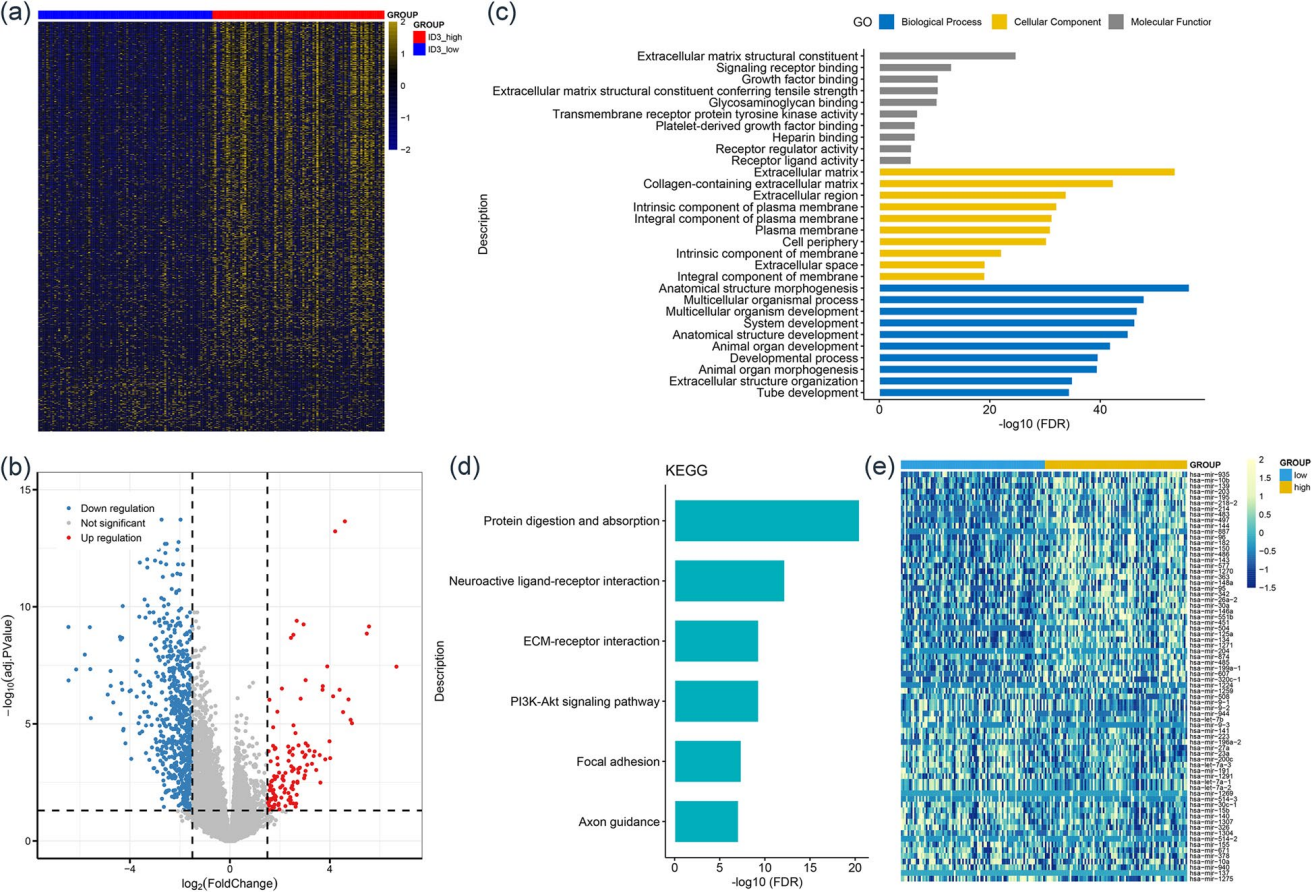
Biological network of aberrant *ID3* expression in AML. **a** Expression heatmap of differentially expressed mRNAs/lncRNAs between low and high *ID3* expression groups in AML (|log2 FC|>1.5, FDR < 0.05 and *P* < 0.05). **b** Volcano plot of differentially expressed mRNAs/lncRNAs between low and high *ID3* expression groups in AML. **c** Gene Ontology (GO) analysis of differentially expressed mRNAs/lncRNAs. **d **Kyoto Encyclopedia of Genes and Genomes (KEGG) analysis of differentially expressed mRNAs/lncRNAs. **e** Expression heatmap of differentially expressed miRNAs between low and high *ID3* expression groups in AML (FDR < 0.05 and *P* < 0.05)

To investigate the biological network caused by aberrant *ID3* expression in AML, we first analyzed the transcriptomes of the two groups of patients (low and high *ID3* expression) from the TCGA dataset. Based on the conditions of |log2 FC|>1.5, FDR < 0.05 and *P* < 0.05, a total of 839 DEGs (706 downregulated and 133 upregulated) between the low and high *ID3* expression groups were identified (Fig. 4a-b and Supplementary Table S4). The top 100 downregulated DEGs, such as *SLIT3* and *ID4*, are reported to have antitumor activities in AML [9, 17, 18]. Moreover, Gene Ontology (GO) and Kyoto Encyclopedia of Genes and Genomes (KEGG) analyses [19–21] revealed that these DEGs are involved in multiple biological processes and the PI3K/AKT signaling pathway (Fig. 4c-d).

We next revealed 72 DEmiRs (38 downregulated and 34 upregulated) between the low and high *ID3* expression groups according to the conditions of FDR < 0.05 and *P* < 0.05) (Fig. 4e and Supplementary Table S4). The top 10 downregulated DEmiRs, including *miR-139*, *miR-195*, *miR-203*, *miR-497* and *miR-144*, are reported to have antitumor effects in AML [22–26]. The top 10 upregulated DEmiRs, such as *miR-196a*, are reported to have protumor effects in AML [27]. Moreover, upregulated DEmiRs (potentially negatively associated with *ID3* expression), such as *miR-326*, have been reported as potential microRNAs that directly target *ID3* [28].

### Validation of ID3 expression and its discriminative capacity in AML patients from our hospital

**Fig. 5:**
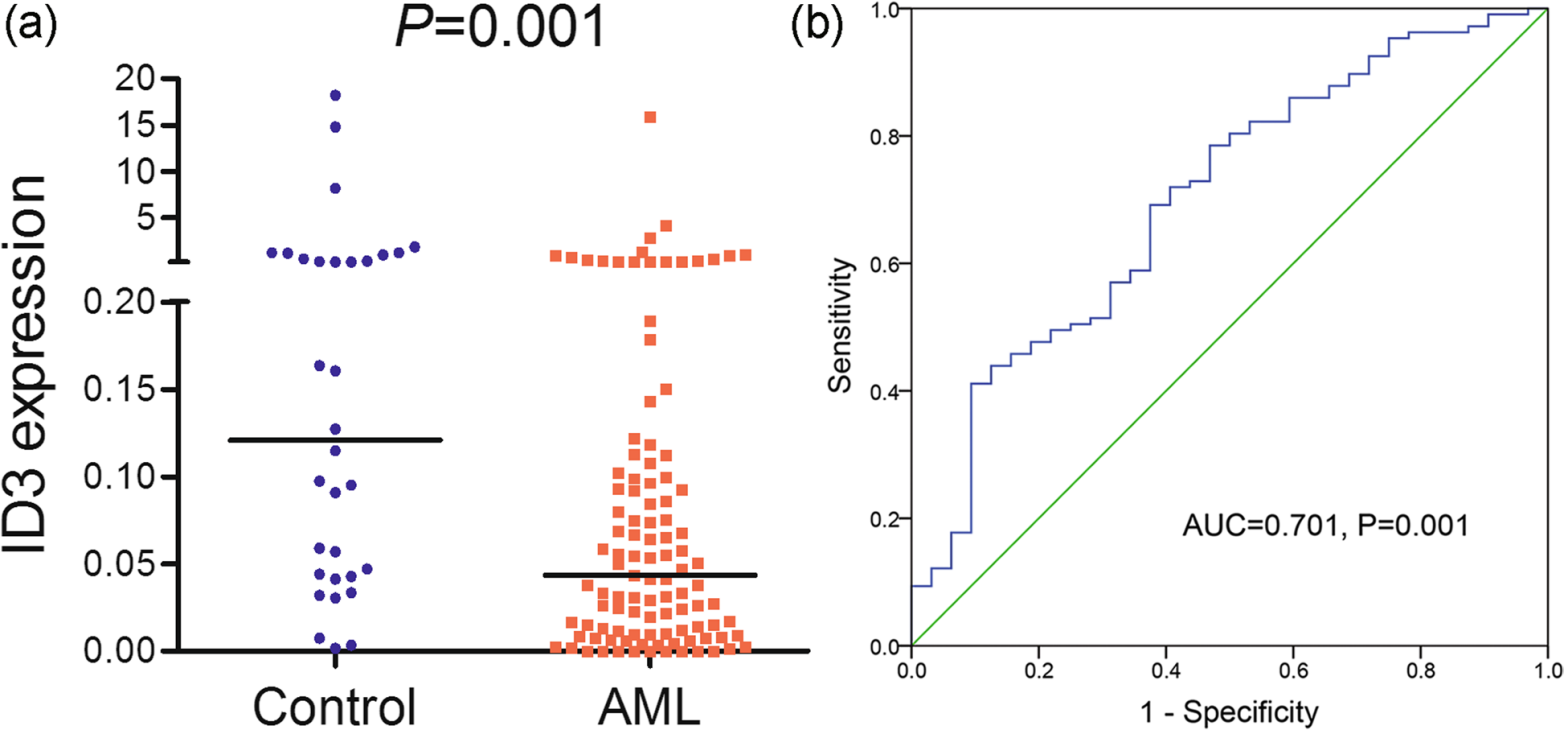
Validation of ***ID3*** expression and its discriminative capacity in AML. **a** The relative expression of *ID3* in AML. **b** ROC curve analysis of *ID3* expression in distinguishing AML controls

Given the results above, we further validated the expression of *ID3* in the BMMNC samples of 107 newly diagnosed AML patients and 32 healthy donors as normal controls from our hospital. The expression of *ID3* was extremely decreased in AML patients compared with normal controls (*P* = 0.001, Fig. 5a). Moreover, ROC analysis indicated that *ID3* expression may serve as a prospective biomarker for discriminating AML patients from controls, with an AUC of 0.701 (95% CI: 0.598–0.805) (*P* = 0.001, Fig. 5b). These results confirmed the low expression pattern of *ID3* in AML and revealed that *ID3* expression might serve as a latent biomarker that is helpful for the diagnosis of AML.

## Discussion

Dysregulation of *ID* gene expression has been revealed in various human cancers including AML, and was also associated with clinical outcome. Recently, Lu et al. using bioinformatics methods revealed that increased expression of *ID1* and *ID2* was correlated with poorer and better survival times, respectively, whereas *ID3* and *ID4* expression was not correlated with survival in lung adenocarcinoma patients [29]. Similarly, abnormal expression of *ID* genes may affect the occurrence and prognosis of lung cancer, and may be associated with cell metabolism and transcriptional regulation by using bioinformatics analysis [30]. These same results were further identified in breast cancer [31]. In the current study, by the bioinformatics analysis, we found that the expression of *ID1* and *ID3* was downregulated in AML, whereas the expression of *ID2* was upregulated. Moreover, only abnormal *ID3* expression may serve as an independent prognostic biomarker in AML and *ID1*/*ID2* expression may independently affect clinical outcome in total AML. Previously, a few studies have reported the prognostic significance of *ID* gene expression in AML. Tang et al. revealed that high *ID1* expression was correlated with adverse prognosis in AML [5]. However, a later study demonstrated that overexpression of *ID1* was not an independent prognostic biomarker in young CN-AML patients [6]. Interestingly, our previous study indicated that overexpression of *ID1* was correlated with higher karyotypic risk classification and served as an independent risk factor in young (age < 60 years) non-M3 patients [7]. Meanwhile, overexpression of *ID2* was a frequent event in patients with AML and predicted poor chemotherapy response and clinical outcome [8]. Conversely, promoter hypermethylation-mediated *ID4* repression was linked to disease progression in MDS and poor prognosis in AML. Altogether, these different results may be attributed to the differences in ethnicity and in AML subtype distribution. Accordingly, further studies are needed to validate the expression and clinical implications of the *ID* genes in AML.

In the present study, we mainly focused on *ID3* expression in AML based on the bioinformatics identification and experimental validation. For the first time, we revealed that *ID3* expression could serve as a prognostic predictor in AML. Notably, it is very interesting that *ID3* could independently affect OS but not DFS by multivariate Cox regression analysis. We deduced that the role of aberrant *ID3* expression in AML survival was not directly mediated by influencing leukemia development but could affect multiple factors that lead to all-cause death in AML. Previously, only May et al. revealed that *ID2* and *ID3* protein expression mirrored granulopoietic maturation and discriminated between acute leukemia subtypes [32]. However, numerous studies have investigated the expression and prognostic value of *ID3* in human solid tumors. Xu et al. demonstrated that *ID3* played a tumor suppressive role in papillary thyroid cancer and impeded metastasis by inhibiting E47-mediated epithelial to mesenchymal transition (EMT) [33]. Huang et al. indicated that *ID3* could enhance the stemness of intrahepatic cholangiocarcinoma by gaining the transcriptional activity of β-catenin and could act as a potential biomarker in predicting response to adjuvant chemotherapeutics [34]. Moreover, *ID3* overexpression was correlated with medulloblastoma seeding and is a poor prognostic factor in medulloblastoma patients [35]. Sharma et al. revealed that *ID1* and *ID3* overexpression alleviated all three cyclin-dependent kinase inhibitors (*CDKN2B*, *-1 A*, and *− 1B*) resulting in a more aggressive prostate cancer phenotype [36]. Expression of *ID1* and *ID3* was increased in human invasive lobular carcinoma compared with invasive ductal carcinoma, associated with poor prognosis uniquely in patients with invasive lobular carcinoma and correlated with the upregulation of angiogenesis and matrisome-related genes [37]. In addition to the above results, several studies have also reported the value of the combination of *ID3* expression with other members in cancer prognosis. For instance, Antonângelo et al. showed that *ID1*, *ID2* and *ID3* coexpression was associated with prognosis in stage I/II lung adenocarcinoma patients treated with surgery and adjuvant chemotherapy [38]. Additionally, *ID1* and *ID3* coexpression was correlated with a poor clinical outcome in patients with locally advanced non-small cell lung cancer treated with definitive chemoradiotherapy [39]. The combined expression of *VPREB3* and *ID3* was used to develop a new helpful tool for the routine diagnosis of mature aggressive B-cell lymphomas [40]. All these results suggested the prognostic value of *ID3* expression in diverse human cancers.

The functional role of *ID3* has also been widely investigated, and was reported to be associated with diverse biological processes such as angiogenesis, apoptosis, cell cycle regulation/proliferation, cell migration/invasion, epithelial-to-mesenchymal transition, stem cell renewal and signaling [3]. Although we did not validate the direct role of *ID3* in AML in this study, we identified the association of *ID3* with PI3K/AKT signaling by bioinformatics methods. Moreover, the association of low *ID3* expression with *FLT3* mutation was also observed in AML patients. Similarly, Chen et al. demonstrated that *miR-212-5p* was involved in the progression of non-small cell lung cancer through the activation of PI3K/Akt signaling pathway by targeting *ID3* [41]. Zhang et al. indicated that Per2 downregulated *ID3* expression via the PTEN/AKT/Smad5 axis to inhibit glioma cell proliferation [42]. Moreover, *ID3* was reported to play a significant role in reversing cisplatin resistance in human lung adenocarcinoma cells by regulating the PI3K/Akt pathway [43]. Accordingly, further functional studies are needed to confirm the direct role of *ID3* in AML biology.

The regulatory mechanism of *ID3* expression was preliminarily studied. Xu et al. demonstrated that hypermethylation of the CpG island at the promoter region of *ID3* was the main contributor to the repression of this gene [33]. In addition, several studies also revealed the regulatory potential of miRNAs. Zhao et al. found that *miR-326* could bind to *ID3*, which accelerated the development of medulloblastoma [28]. Moreover, high *ID3* expression by silencing *miR-212-5p* expression suppressed the activity of the PI3K/Akt signaling pathway and consequently promoted apoptosis and inhibited proliferation in lung cancer cells [41]. Herein, we also observed the association of *ID3* with several miRNAs such as *miR-1259*, *miR-508*, *miR-9*, *miR-944*, *let-7b*, *miR-141*, and *miR-223*. However, only *miR-326* was confirmed by previous studies [28]. Accordingly, further studies are needed to confirm the direct association of *ID3* with these miRNAs.

## Conclusion

In summary, the bioinformatics analysis and experimental verification demonstrate that low *ID3* expression independently affects OS and DFS in patients with CN-AML, which might be seen as a potential prognostic indicator in CN-AML.

## Supplementary Information

**Additional file 1:** **Table S1.** Clinic-pathologic characteristics of AML in our research cohort.

**Additional file 2**: **Table S2.** Cox regression univariate and multivariate analysis of variables for disease free survival in AML patients.

**Additional file 3:** **Table S3.** Cox regression univariate and multivariate analysis of variables for disease free survival in AML patients.

**Additional file 4:** **Table S4.** Different expressed genes/miRNAs between low and high ID3 expression groups.

## Data Availability

The datasets analyzed in this study are available in the following open access repositories: cBioPortal (http://www.cbioportal.org/); TCGA (https://portal.gdc.cancer.gov/) and GEPIA (http://gepia.cancer-pku.cn/).

## Abbreviations

ID: Inhibitor of Differentiation/DNA binding
AML: Acute myeloid leukemia
CN-AML: Cytogenetically normal AML
BM: Bone marrow
MICM: Morphology, immunology, cytogenetics and molecular biology
TCGA: The Cancer Genome Atlas
BMMNCs: BM mononuclear cells
FDR: False discovery rate
DFS: Disease-free survival
OS: Overall survival
AUC: Area under the ROC curve
ROC: Receiver operating characteristic
WBC: White blood cell
PB: Peripheral blood
FAB: French–American–British
DEGs/DEmiRs: Differentially expressed genes/miRNAs
GO: Gene Ontology
KEGG: Kyoto Encyclopedia of Genes and Genomes
EMT: Epithelial to mesenchymal transition
