# Supplementary material for: Comprehensive analysis of ID genes reveals the clinical and prognostic value of ID3 expression in acute myeloid leukemia using bioinformatics identification and experimental validation
Source: BMC Cancer. 2022 Nov 29;22:1229. doi: 10.1186/s12885-022-10352-6 (PMC9707109; doi:10.1186/s12885-022-10352-6)
Supplement: Supplementary file 1 — Additional file 1: Table S1. Clinic-pathologic characteristics of AML in our research cohort. [file 12885_2022_10352_MOESM1_ESM.docx]

**Table S1. Clinic-pathologic characteristics of AML in our research cohort**

| Patient's parameters | Total (n=107) |
| --- | --- |
| Sex, male/female | 61/46 |
| Median age, years (range) | 57 (18-87) |
| Median WBC, ×10^9^/L (range) | 15.9 (0.3-528.0) |
| Median hemoglobin, g/L (range) | 76 (32-138) |
| Median platelets, ×10^9^/L (range) | 39 (3-447) |
| BM blasts, % (range)^#^ | 39 (1.0-99.0) |
| FAB classifications |  |
| M0 | 1 |
| M1 | 6 |
| M2 | 39 |
| M3 | 21 |
| M4 | 29 |
| M5 | 9 |
| M6 | 2 |
| Karyotypes |  |
| normal | 51 |
| t(8;21) | 7 |
| t(15;17) | 21 |
| +8 | 2 |
| -5/5q- | 3 |
| -7/7q- | 1 |
| complex | 11 |
| others | 6 |
| No data | 5 |
| Risks (cytogenetic) |  |
| Favorable | 28 |
| Intermediate | 59 |
| Poor | 15 |
| No data | 5 |
| Gene mutations^*^ |  |
| *CEBPA* (+/-) | 10/89 |
| *NPM1* (+/-) | 9/90 |
| *FLT3*-ITD (+/-) | 12/87 |
| *CKIT* (+/-) | 4/95 |
| *KRAS* (+/-) | 2/97 |
| *NRAS* (+/-) | 4/95 |
| *IDH1* (+/-) | 1/98 |
| *IDH2* (+/-) | 5/94 |
| *DNMT3A* (+/-) | 7/92 |
| *U2AF1* (+/-) | 4/95 |
| *SRSF2* (+/-) | 6/93 |
| CR (+/-) | 44/61 |

AML: acute myeloid leukemia; WBC: white blood cells; BM: bone marrow; FAB: French-American-British classification; CR: complete remission; ^#^BM blasts less 20% diagnosed with AML usually with chromosome abnormalities such as t(15;17) and t(8;21); ^*^gene mutations are hotspot sites mutations.
