# Supplementary material for: Comprehensive analysis of ID genes reveals the clinical and prognostic value of ID3 expression in acute myeloid leukemia using bioinformatics identification and experimental validation
Source: BMC Cancer. 2022 Nov 29;22:1229. doi: 10.1186/s12885-022-10352-6 (PMC9707109; doi:10.1186/s12885-022-10352-6)
Supplement: Supplementary file 2 — Additional file 2: Table S2. Cox regression univariate and multivariate analysis of variables for disease free survival in AML patients. [file 12885_2022_10352_MOESM2_ESM.docx]

**Table S2. Cox regression univariate and multivariate analysis of variables for disease free survival in AML patients**

| Variables | Univariate analysis | | Multivariate analysis | |
| --- | --- | --- | --- | --- |
|  | Hazard ratio (95% CI) | *P* value | Hazard ratio (95% CI) | *P* value |
| Total AML | | | | |
| *ID1* expression | 1.327 (0.919-1.918) | 0.131 | 1.488 (1.013-2.186) | 0.043 |
| *ID2* expression | 1.428 (0.987-2.068) | 0.059 | 1.392 (0.961-2.106) | 0.080 |
| *ID3* expression | 0.707 (0.488-1.023) | 0.066 | 0.636 (0.432-0.936) | 0.022 |
| *ID4* expression | 0.778 (0.538-1.124) | 0.181 | 0.796 (0.541-1.169) | 0.245 |
| CN-AML | | | | |
| *ID1* expression | 1.059 (0.619-1.814) | 0.834 | - | - |
| *ID2* expression | 1.125 (0.658-1.922) | 0.667 | - | - |
| *ID3* expression | 0.536 (0.295-0.973) | 0.041 | 0.536 (0.295-0.973) | 0.041 |
| *ID4* expression | 0.591 (0.342-1.021) | 0.059 | 0.663 (0.379-1.162) | 0.151 |

AML: acute myeloid leukemia; CN-AML: cytogenetically normal AML; CI: confidence interval.
