# Supplementary material for: Comprehensive analysis of ID genes reveals the clinical and prognostic value of ID3 expression in acute myeloid leukemia using bioinformatics identification and experimental validation
Source: BMC Cancer. 2022 Nov 29;22:1229. doi: 10.1186/s12885-022-10352-6 (PMC9707109; doi:10.1186/s12885-022-10352-6)
Supplement: Supplementary file 3 — Additional file 3: Table S3. Cox regression univariate and multivariate analysis of variables for disease free survival in AML patients. [file 12885_2022_10352_MOESM3_ESM.docx]

**Table S3. Cox regression univariate and multivariate analysis of variables for disease free survival in AML patients**

| Variables | Total AML | | | | CN-AML | | | |
| --- | --- | --- | --- | --- | --- | --- | --- | --- |
|  | Univariate analysis | | Multivariate analysis | | Univariate analysis | | Multivariate analysis | |
|  | HR (95% CI) | *P* | HR (95% CI) | *P* | HR (95% CI) | *P* | HR (95% CI) | *P* |
| Age | 1.035 (1.021-1.049) | <0.001 | 1.029 (1.014-1.044) | <0.001 | 1.020 (1.001-1.038) | 0.035 | 1.012 (0.991-1.033) | 0.273 |
| WBC | 1.005 (1.001-1.009) | 0.008 | 1.006 (1.002-1.011) | 0.004 | 1.005 (1.000-1.010) | 0.033 | 1.003 (0.998-1.009) | 0.208 |
| Molecular risks | 1.824 (1.423-2.339) | <0.001 | 1.930 (1.440-2.586) | <0.001 | 1.491 (0.638-3.489) | 0.357 | - | - |
| Treatment regimen | 0.584 (0.399-0.855) | 0.006 | 0.512 (0.335-0.784) | 0.002 | 0.589 (0.343-1.011) | 0.055 | 0.476 (0.270-0.839) | 0.010 |
| *FLT3* mutation^#^ | 1.328 (0.887-1.989) | 0.168 | 1.500 (0.962-2.338) | 0.073 | 1.454 (0.840-2.515) | 0.181 | 1.471 (0.801-2.704) | 0.214 |
| *NPM1* mutation | 1.183 (0.788-1.774) | 0.417 | - | - | 1.047 (0.613-1.789) | 0.866 | - | - |
| *TP53* mutation | 3.456 (1.954-6.115) | <0.001 | 2.474 (1.277-4.791) | 0.007 | 3.003 (0.405-22.291) | 0.282 | - | - |
| *CEBPA* mutation^&^ | 1.054 (0.533-2.085) | 0.879 | - | - | 1.074 (0.459-2.516) | 0.869 | - | - |
| *ASXL1* mutation | 1.934 (0.613-6.097) | 0.260 | - | - | 10.795 (1.328-87.751) | 0.026 | 18.562 (2.110-163.265) | 0.008 |
| *RUNX1* mutation | 0.999 (0.710-1.405) | 0.995 | - | - | 1.243 (0.494-3.128) | 0.644 | - | - |
| *ID1* expression | 1.327 (0.919-1.918) | 0.131 | 1.252 (0.828-1.894) | 0.287 | 1.059 (0.619-1.814) | 0.834 | - | - |
| *ID2* expression | 1.428 (0.987-2.068) | 0.059 | 1.044 (0.694-1.570) | 0.837 | 1.125 (0.658-1.922) | 0.667 | - | - |
| *ID3* expression | 0.707 (0.488-1.023) | 0.066 | 0.784 (0.514-1.193) | 0.256 | 0.536 (0.295-0.973) | 0.041 | 0.523 (0.279-0.980) | 0.043 |
| *ID4* expression | 0.778 (0.538-1.124) | 0.181 | 0.674 (0.449-1.012) | 0.057 | 0.591 (0.342-1.021) | 0.059 | 0.529 (0.294-0.952) | 0.034 |

AML: acute myeloid leukemia; CN-AML: cytogenetically normal AML; HR: hazard ratio; CI: confidence interval; WBC: white blood cells. Variables including age (continuous variables), WBC (continuous variables), treatment regimen (with transplantation vs. without transplantation), molecular risks (good, intermediate, poor and unknown; classified by the 2017 European LeukemiaNet classification), *FLT3*/*NPM1*/*TP53* mutation (wild type vs. mutant) and *ID1/2/3/4* expression (low vs. high). Multivariate analysis includes variables with *P*<0.200 in univariate analysis. ^#^*FLT3* mutation indicates both *FLT3-ITD* (high and low ratios) and *FLT3-TKD* mutations. ^&^*CEBPA* mutation indicates both mono- and bi-allelic *CEBPA* mutation.
